# Toward QbD Process Understanding on DNA Vaccine Purification Using Design of Experiment

**DOI:** 10.3389/fbioe.2021.657201

**Published:** 2021-05-12

**Authors:** Lalintip Hocharoen, Sarawuth Noppiboon, Panit Kitsubun

**Affiliations:** ^1^Bioprocess Research and Innovation Centre (BRIC), National Biopharmaceutical Facility (NBF), King Mongkut’s University of Technology Thonburi (KMUTT), Bangkok, Thailand; ^2^Biochemical Engineering and System Biology Research Group (IBEG), National Center for Genetic Engineering and Biotechnology (BIOTEC), National Science and Technology Development Agency (NSTDA), Bangkok, Thailand

**Keywords:** QbD, process understanding, DNA vaccine purification, Design of Experiment, tolerance study

## Abstract

DNA vaccines, the third generation of vaccines, are a promising therapeutic option for many diseases as they offer the customization of their ability on protection and treatment with high stability. The production of DNA vaccines is considered rapid and less complicated compared to others such as mRNA vaccines, viral vaccines, or subunit protein vaccines. However, the main issue for DNA vaccines is how to produce the active DNA, a supercoiled isoform, to comply with the regulations. Our work therefore focuses on gaining a process understanding of the purification step which processes parameters that have impacts on the critical quality attribute (CQA), supercoiled DNA and performance attribute (PA), and step yield. Herein, pVax1/lacZ was used as a model. The process parameters of interest were sample application flow rates and salt concentration at washing step and at elution step in the hydrophobic interaction chromatography (HIC). Using a Design of Experiment (DoE) with central composite face centered (CCF) approach, 14 experiments plus four additional runs at the center points were created. The response data was used to establish regression predictive models and simulation was conducted in 10,000 runs to provide tolerance intervals of these CQA and PA. The approach of this process understanding can be applied for Quality by Design (QbD) on other DNA vaccines and on a larger production scale as well.

## Introduction

DNA vaccines become prominent for use against several diseases including VCL-CB01, a candidate for cytomegalovirus (CMV)-DNA vaccine (Wloch et al., 2008), GX188E and VGX-3100 for Human Papillomavirus (HPV) (Cheng et al., 2018), a prime/boost of DNA.Mel3 with MVA.Mel3 for advanced metastatic melanoma cancer treatment (Dangoor et al., 2010), a pDERMATT for vaccination against melanoma (Quaak et al., 2008), and recently the INO-4800 for SARS-CoV2 in COVID-19 vaccine development (Smith et al., 2020). However, these candidate human vaccines remain in clinical trial studies. The highlighted advantages of DNA vaccines over other platforms, such as mRNA, protein-based, or monoclonal antibody or viral vaccines, are that DNA vaccines are more stable, have fewer adverse effects, and have a less complicated manufacturing process (Cai et al., 2010; Rauch et al., 2018; Liu, 2019; Almeida et al., 2020). The cell expansion uses *Escherichia coli* cells which grow faster and easier than mammalian cell lines, which are used in some protein-based and monoclonal antibody production (Liu, 2019; Tripathi and Shrivastava, 2019). The cell lysis step frequently undergoes an alkaline cell lysis followed by high molecular weight RNA precipitation using chemical reagents such as Calcium chloride. Since DNA vaccines are highly negatively charged, often anion exchange chromatography is selected as the first capture step. Other purification approaches have also been extensively performed, such as one step purification using *O*-Phospho-l-tyrosine resin in purifying the 6.07 kbp pcDNA3-FLAG-p53 plasmid (Valente et al., 2019), arginine monolith for HPV-16 E6/E7 plasmid-based vaccine (Almeida et al., 2015), and pyridine-modified methacrylate monolithic column (Cardoso et al., 2015). When required, alternative polishing steps using other types of media may be needed to achieve product quality as desired. To achieve this, a systematic approach, Quality by Design (QbD) developed by Juran (1992), has been introduced to pharmaceutical industries.

Quality by Design emphasizes the product and process understanding, design space, control strategies, and continual improvement strategy using several tools such as prior knowledge, risk assessment, Design of Experiment (DoE), and Process Analytical Technology (PAT) (ICH, 2009; Jiang et al., 2010; McCurdy, 2011; Yu et al., 2014; Dey and Chowdhury, 2018). This aligns well with current regulatory requirements, but it is of the utmost importance to characterize a production process and the impact of operational parameters on the product and process quality attributes. When the process is developed and ready to scale up to a pilot study, it is important to demonstrate process robustness and identify critical process parameters, so called process characterization studies, before process industrialization and process transfer to manufacturing (Helling and Strube, 2012). Process optimization is a part of this characterization process to ensure that the product meets specifications. The U.S. FDA recommends that the fraction of plasmid in supercoiled conformation be included in the bulk release criteria, and that a minimum specification for supercoiled plasmid content is established and is preferably >80%. This is due to higher efficacy of supercoiled plasmid over other isoforms (Cupillard et al., 2005; U.S. FDA, 2007; Valente et al., 2018; Azevedo et al., 2019).

We presented herein a systematic approach onto process understanding of a hydrophobic interaction chromatography (HIC)-step purification of DNA vaccine using pVax1/lacZ and Design of Experiment (DoE) as a tool. HIC-step is used as a polishing step to isolate isoform of the DNA after other impurities, such as RNA, have been removed by anion exchange chromatography. This two-step purification may result in a longer process time, however, with convective flow monolithic stationary phase, high flow rate can be applied, thus possibly compensating for the process time (Urthaler et al., 2005). The process parameters of interest were selected based on our prior knowledge and risk assessment that the application flow rate theoretically influences the binding capacity while the salt concentrations play an important role as an antichaotropic agent used in washing impurities and eluting the products. To assess the process performance of these three parameters, percentage of step field (%Step yield) was evaluated as process attribute (PA) and percentage of step coil (%SC) content was monitored as a critical quality attribute (CQA). The total of eighteen experimental runs were designed by using central composite face centered (CCF), as this model covers a larger design space, provides the smallest predicted error of center runs, giving better prediction models for our study, and requires only three level settings of parameters, making it an undemanding experiment (Box and Wilson, 1951; Ferreira et al., 2007; Zhang and Xiaofeng, 2009; Montgomery, 2017). The relationships presented here were expected to provide predictive models used in QbD and potentially establish a design space systematic approach that could be applied to other biopharmaceutical productions.

## Materials and Methods

### Materials

*Escherichia coli* DH5α [F–Φ 80dlacZΔM15 Δ(lacZYA-argF) U169 recA1 endA1 hsdR17(rk-, mk+) phoA supE44 λ-thi-1 gyrA96 relA1], pVax1/lacZ plasmid, DNA staining reagent, and SYBR^®^ Gold Nucleic Acid Gel Stain were purchased from Thermo Fisher Scientific. LB medium and yeast extract were purchased as dehydrated powers from BD Bacto while other chemicals, such as reagents used in fermentation, cell lysis, or in buffer system, were all from Merck. Consumables such as liquid filters were from Sartorius while Tangential Flow Filtration (TFF) cassette was from Pall. 8-mL DEAE, 8-mL C4, and 0.3 mL analytical columns were from BIA separations. Statistical analysis software was JMP Pro software from SAS Institute Inc.

### Cell Cultivation

*Escherichia coli* DH5α [F–Φ 80dlacZΔM15 Δ(lacZYA-argF) U169 recA1 endA1 hsdR17(rk-, mk+) phoA supE44 λ-thi-1 gyrA96 relA1] containing pVax1/lacZ was used for this study. The first cultivation began with 1% inoculation in 100 mL LB medium and was incubated at 30°C and 200 rpm (Innova 43R, New Brunswick) until its optical density (OD_600_) reached 0.5–1.0. This culture was transferred to a 3 L (working volume) in a fermenter (R’ALF, BioEngineering). The fermenter’s cultivation media contained 3 g/L KH_2_PO_4_, 6 g/L Na_2_HPO_4_, 2 g/L NH_4_Cl, 1g/L MgSO_4_, 30 g/L yeast extract, 5 g/L glycerol, and 100 mg/L Thiamine HCl.

Batch fermentation began with setpoints at 30°C, pH 7, 30%DO, and 1 vvm air flow rate. Cells were grown until the OD_600_ reached 15 then the fed-batch was performed by adding glycerol exponentially. This exponential glycerol feed was calculated from the equation shown below;

$$\text{F}=\frac{\mu \,X_{0}\,V\,e^{\mu \,t}}{S_{0}\,Y_{x/s}}$$

where,

F is exponential feed rate (L/h),
μ is a specific growth rate which was fixed at 0.15 h^–1^ (Huber et al., 2009),
X_0_ is cell concentration (g dry weight/L) which herein was 7.5 (Folsom et al., 2014),
V is medium working volume which was 3 L,
S_0_ is substrate concentration (g/L) which was 600 g/L,
and Y_*x/s*_ is a yield coefficient which herein was 0.5.

The feed rate was then converted to peristaltic pump % where the pump was equipped in the fermenter controller. The cultivation was ended when the cells were in stationary phase. Cells were harvested by centrifugation at 7,500 × *g* for 0.5 h (Lynx6000, Thermo Fisher Scientific). Wet cell paste (WCP) was stored at −20°C for future use.

### Plasmid DNA Recovery

Wet cell paste then underwent chemical lysis starting with 10% w/v cell resuspension in Tris-EDTA buffer (50 mM Tris, 2.5 mM EDTA pH 8.0), alkaline cell lysis with 200 mM NaOH and 1% SDS, and finally neutralization with 3 M potassium acetate. The ratio of these solution was 1:1:1. Cell lysis solution was centrifuged at 14,000 × *g* for 0.5 h (Lynx6000, Thermo Fisher Scientific) and the supernatant was collected for a further RNA precipitation step which was performed by adding CaCl_2_ to 1 M final concentration. RNA precipitants were removed by centrifugation at 12,000 × *g* for 0.5 h. The pDNA supernatant was further clarified through 5 μm depth filter (Sartopure PP3, Sartorius) and 0.8 + 0.2 μm (Sartopore 2 XLG, Sartorius) in series. Buffer exchange to Tris-EDTA buffer (50 mM Tris, 10 mM EDTA pH 7.2) containing 0.6 M NaCl was conducted using TFF (AkTA flux 6, GE healthcare) with 50 kD MWCO TFF cassette (Omega 50 kD Centramate T-series, Pall). The pDNA clarified lysate was concentrated to 0.5 L then harvested from TFF and stored at −20°C until use.

### Plasmid DNA Purification

Two-step chromatography (AkTA Pure 150, GE Healthcare) for pDNA purification using 8-mL anion exchange chromatography (AIEX) column (CIMmultus DEAE-8, BIA separations) and 1-mL hydrophobic interaction chromatography (HIC) column (CIMmultus C4 HLD-1, BIA separations) was performed at room temperature. Buffer systems for the first chromatography were Tris-EDTA pH 7.2 containing no salt for equilibration step, 0.6 M NaCl for washing impurity step, and 1 M NaCl for DNA elution step.

This elution was collected and checked for pDNA concentration by A_260_ method (BioSpectrometer Kinetic, Eppendorf) in order to calculate % step yield for HIC step. The AIEX elution was further incubated with 3 M ammonium sulfate for 1 h before being loaded onto the HIC column which was equilibrated with Tris-EDTA buffer pH 7.2, washed, and eluted with Tris-EDTA buffer containing various concentration of ammonium sulfate [(NH_4_)_2_SO_4_] as described in session Design of Experiment.

### Plasmid DNA Qualification and Quantification

A_260_ method (McGown, 2000; Stephenson, 2003) was used as a quantification method of purified nucleic acids. The AIEX elution fraction was quantified as the total nucleic acid which was then loaded onto HIC column. The HIC elution fraction was checked for pDNA concentration. Therefore, step yield was calculated based on amount of total nucleic acid loaded onto HIC column and its elution fraction.

pDNA qualification was determined by high performance liquid chromatography (HPLC) (SPD-20A, Shimadzu) using 0.3 mL AIEX column (CIMacTM pDNA analytical column, BIA separations). The DNA quantification using HPLC technique was adapted from Validation of an analytical method using an anion-exchange monolithic column for the assessment of supercoiled plasmid DNA (Mota, 2012) in which 10 μL of each sample was loaded onto the column and the flow rate was set at 1 mL/min. By keeping the constant volume loaded to HPLC, the area under elution peaks from each sample was directly calculated and compared among different experimental runs. HPLC mobile phases consisted of buffer A (Tris-EDTA buffer pH 8 containing 0.6 M NaCl) and buffer B (Tris-EDTA buffer pH 8 containing 1 M NaCl). The equilibration and sample application steps were set to 85% buffer A mixed with 15% buffer B. The gradient was then set to 20% buffer B over 3.5 min to elute non-supercoiled forms, such as open circular (OC) pDNA, and followed by a linear gradient from 30 to 45% over 3 min to elute supercoiled pDNA. The UV detector at 260 nm was monitored and the chromatogram is shown in the Supplementary Material. The %SC content was calculated based on the areas under the elution peaks.

Agarose gel electrophoresis (Bio-Rad) was performed to check impurities in each process step. 0.7% agarose gel was used and stained with SYBR^®^ Gold Nucleic Acid Gel Stain (Thermo Fisher Scientific) and then visualized by gel imager (Bio-Rad).

### Design of Experiment and Tolerance Interval Study

Parameters of interest were from HIC purification step which were flow rate of sample application and concentrations of ammonium sulfate to wash other isoforms of pDNA and to elute supercoiled pDNA. These were chosen to perform process optimization as the HIC step is vital on pDNA isoform isolation where the active form to be used as DNA vaccine is in a supercoiled form (U.S. FDA, 2007). The experiment was designed using response surface method with CCF design in which 18 experimental runs were created. Four replicate runs at the center points were also included in order to better estimate the error of experiments. Table 1 demonstrates all 18 experimental runs for process optimization in HIC purification step. Statistical analysis was performed using JMP Pro software (SAS Institute Inc.). The model prediction was established based on a model selection using criteria of combined corrected Akaike Information Criterion (AICc) and the Bayesian Information Criterion (BIC) where the lower AICc or BIC indicate better model prediction. Thus, the models with ΔAICc less than or equal to 4 and ΔBIC less than or equal 2 were selected (Burnham and Anderson, 2004; Ward, 2008; Mangan et al., 2017; Hocharoen et al., 2020). A further consideration was the coefficient of determination (R^2^). The prediction profiler function was then used for process optimization. The optimization was expected to provide an understanding of what factors mainly influence the HIC purification step for achieving a qualified product. Furthermore, a Monte Carlo simulation with random variation derived from root mean square error (RMSE) of the obtained predictive models was performed in 10,000 runs for a tolerance interval (TI) study. This TI can then be set for the action and alert limit for process parameters and product specifications for critical quality attributes for production on a larger scale.

**TABLE 1:** Central composite face centered (CCF) and experimental data responses.

**Run**	**Variable level**			**% pDNA Step yield**	**%SC pDNA**
	**Flow rate (mL/min)**	**(NH_4_)_2_SO_4_ concentration at washing step (M)**	**(NH_4_)_2_SO_4_ concentration at elution step (M)**		
1	5.5	1.53	0.36	78.05	100.00
2	5.5	1.53	0.44	78.05	97.05
3	4.5	1.7	0.4	79.68	96.00
4	5	1.7	0.4	79.71	97.07
5	4.5	1.53	0.44	80.43	97.43
6	5.5	1.87	0.44	86.45	89.59
7	4.5	1.87	0.44	86.09	88.34
8	5.5	1.87	0.36	86.03	88.64
9	5.5	1.7	0.4	80.51	97.03
10	5	1.87	0.4	86.21	89.03
11	4.5	1.53	0.36	80.15	98.54
12	5	1.7	0.36	80.54	99.56
13	4.5	1.87	0.36	86.60	89.35
14	5	1.7	0.4	76.74	99.44
15	5	1.7	0.44	79.67	98.45
16	5	1.53	0.4	79.29	99.29
17	5	1.7	0.4	80.29	99.58
18	5	1.7	0.4	80.01	99.59

## Results and Discussion

**FIGURE 1:**
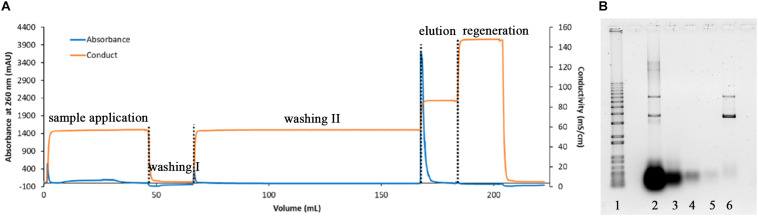
Chromatogram of the DEAE capture step **(A)** and respective agarose electrophoresis **(B)** where lane 1 is 1 kb plus DNA ladder, lane 2 lysate, lane 3 after RNA precipitation, lane 4 AIEX flow through fraction, lane 5 AIEX washing II fraction, and lane 6 AIEX elution fraction.

The fermentation of *E. coli* producing pVax1/lacZ was successfully carried out in 3 L semi-defined media with fed-batch strategy providing sufficient materials for downstream processing. Using alkaline lysis, 100 g of wet cell pastes were lysed, followed by CaCl_2_ precipitation, centrifugation, and a series of filtrations. The mixture then proceeded to buffer exchange and a concentration to 0.5 L using TFF prior to anion exchange chromatography. This first purification step was a capture step where all anion components were attached onto the column and salt ionic strength was increased in proportion to its concentration; the product was then eluted (Stadler et al., 2004; Sun et al., 2013; Silva-Santos et al., 2017). In our experiment, our pDNA was eluted with Tris-EDTA buffer containing 1 M NaCl while impurities such as remaining RNA came out with lower salt concentrations at 0.6 M, as displayed in Figure 1A. The anion exchange chromatography elution fraction was collected and checked for the product concentration with UV/Vis spectrophotometer. It was 150 μg/mL with approximately 80% SC content based on the agarose gel electrophoresis, shown in Figure 1B, where the high molecular weight RNA was reduced after CaCl_2_ precipitation and the remaining RNA seemed to be mostly washed out in AIEX washing step.

However, the regulatory aspect regarding the supercoiled form of DNA vaccine requires more than 80% (U.S. FDA, 2007), so the isolation of isoform of pDNA has to be further conducted. Therefore, hydrophobic interaction chromatography was chosen to isolate different isoforms of pDNA because different isoforms have distinct hydrophobicity properties. The supercoiled pDNA has the hydrophobic bases well packed inside the double strands, preventing exposure with the HIC ligand while the open circular or linear pDNA are more relaxed in structures leading to higher exposure of hydrophobic bases and stronger interaction to HIC media. Using the butyl group (C4) as HIC ligands on the Convective Interaction Media (CIM) and primarily hydrophilic of all pDNA isoforms, a high concentration of salt is required for pDNA to bind onto the HIC column in the first place and then a decrease of salt gradient is used to desorb linear, open circular, and supercoiled isoforms sequentially. This was also supported by a finding from the study by Azevedo et al. on interaction of different types of resin and supercoiled DNA as well as Roettger and colleagues’ work on adsorption phenomena in hydrophobic interaction chromatography (Roettger et al., 1989; Azevedo et al., 2019). With this prior knowledge we selected the salt concentrations at the washing step and elution step as our process parameters. Ammonium sulfate [(NH_4_)_2_SO_4_] is widely used in commercial processes as an antichaotropic agent for HIC chromatography. Various concentrations have been employed in other pDNA studies, so herein we did a literature review and set the process value at 10% variation from the center points which were 1.7 M and 0.4 M for washing and elution steps, respectively. Moreover, we performed a risk assessment together with prior knowledge on the flow rate which theoretically impacts the binding capacity due to the residence time factor (Bergander et al., 2008). As a result, we selected the flow rate during sample application as one of our interest parameters. Our aim was to gain an understanding of this HIC step and to be able to do a scaled up process, hence, five column volume (CV)/minute, as recommended from the BIA separations, was initially chosen as a center point and the range for CCF studies were within 10% from the center point.

CCF was chosen over other central composite designs as it has the smallest predicted error of center runs, giving more robustness for the center runs (Zhang and Xiaofeng, 2009). CCF requires only three level settings of the parameters, making it a manageable design to execute. The model consists of 2^*k*^ +2k + C experimental runs where k is the number of process parameters and C is the number of replications at the center point (Box and Wilson, 1951; Montgomery, 2017). Thus, with the three process parameters mentioned above, the number of runs would be 18 including four replicates at the center point. This replication run is for a better estimation on the error of experiment. The responses we measured were pDNA concentration from HIC elution, which was converted to %Step yield, and %SC pDNA in the elution which was obtained from HPLC runs (see Supplementary Material for HPLC chromatograms).

**FIGURE 2:**
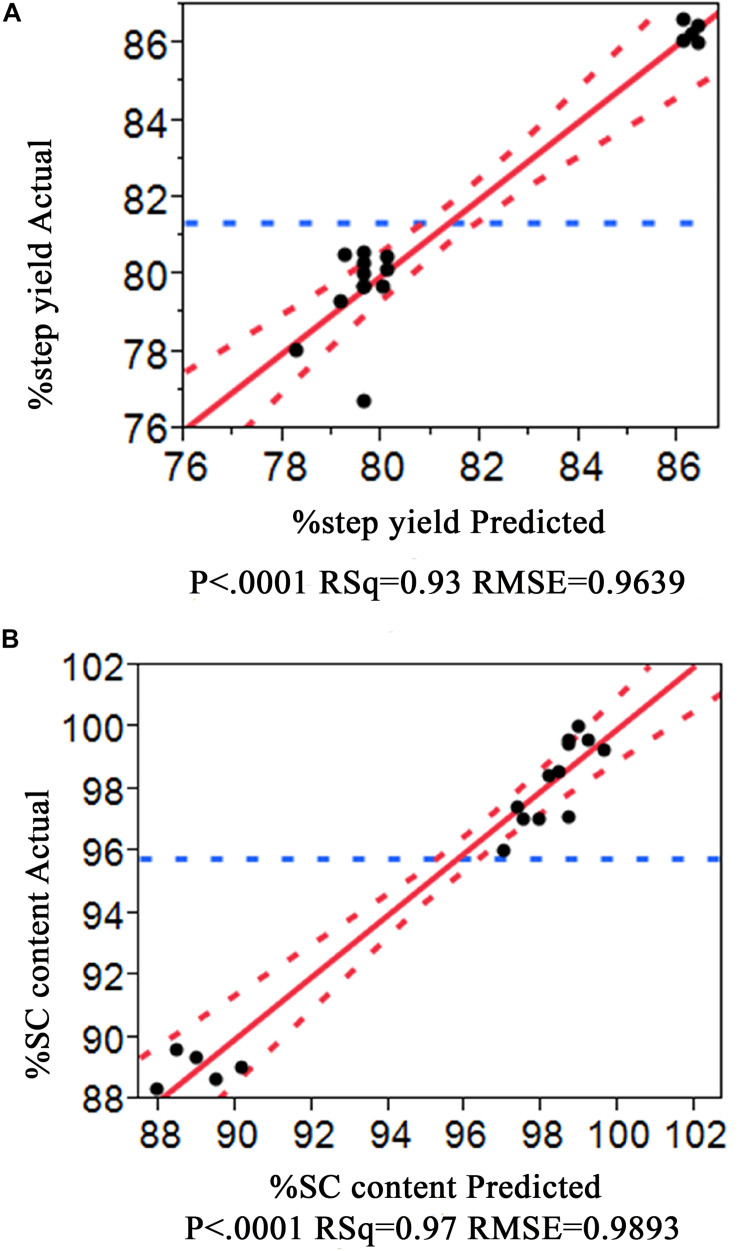
Prediction plot for %Step yield **(A)** and for %SC pDNA **(B)**.

The experiment results are shown in Table 1 where a substantial variation ranges from 79.29 to 86.60 for %Step yield and 88.34 −100 for %SC pDNA. Interestingly, all HIC purification conditions performed demonstrated higher %SC pDNA than what is required from regulations. Thus, the optimized process from our data would provide the maximized responses of %Step yield and %SC content. These data were fitted using JMP Pro Software and the prediction model was created using all possible models with combined AICc and BIC, where the models with ΔAICc less than or equal to 4 and ΔBIC less than or equal 2 were selected. These AICc and BIC calculations measure the model performance in which the smaller values indicate better model prediction (Burnham and Anderson, 2004). After the models were chosen, the *R*^2^ was evaluated. Generally higher *R*^2^ ranging between 0 and 1 means the model better fits the data. Our statistical results showed that the *R*^2^ of models corresponding to %Step yield was 0.93 and %SC was 0.97, indicating that our selected models nicely aligned with the data as also appeared in the actual and predicted plots in Figure 2A for %Step yield and Figure 2B for %SC pDNA.

As a result, the 4-term model for %Step yield and 5-term model for %SC were selected and their corresponding analysis of variance (ANOVA) were presented in Tables 2, 3, respectively. The probability value (*p*-value) for these two models were lower than 0.05, confirming that the model data are statistically significant. Considering the Lack of fit which relies on the ability to estimate the response’s variance by using an estimate that has no dependency on the model, the Lack of fit *F*-value to %Step yield was 0.7429 and to %SC pDNA was 0.9866, implying that the Lack of fit was not significant relative to their corresponding pure error, underlying that these can be used for predictive model estimation.

**TABLE 2:** Regression analysis of predicted model for %Step yield.

**Source**	**DF**	**Sum of squares**	**Mean square**	**F Ratio**	**Prob > F**
Model	4	171.62622	42.9066	46.1863	<0.0001*
Error	13	12.07756	0.929		
C. total	17	183.70378			
Source	DF	Sum of squares	Mean square	F Ratio	Prob > F
Lack of fit	4	2.163972	0.54099	0.4911	0.7429
Pure error	9	9.913583	1.10151		
Total error	13	12.077555			


**Term**		**Estimate**	**Std error**	***t* Ratio**	**Prob > |t|**

Intercept		79.64375	0.340119	233.71	< −0.000*
Flow rate		−0.386	0.304802	−1.27	0.2276
Salt concentration at washing step		3.541	0.304802	11.62	<0.0001*
Flow rate*salt concentration at washing step		0.53375	0.340779	1.57	0.1413
Salt concentration at washing step*salt concentration at washing step		3.09125	0.457203	6.76	<0.0001*

*Identified variable with a significant effect on response (*p*-value < 0.05).

**TABLE 3:** Regression analysis of predicted model for %SC pDNA.

**Source**	**DF**	**Sum of squares**	**Mean square**	**F Ratio**	**Prob > F**
Model	5	328.9396	65.7874	67.215	<0.0001*
Error	12	11.74512	0.9788		
C. total	17	340.682			
Source	DF	Sum of squares	Mean square	F Ratio	Prob > F
Lack of fit	9	7.167719	0.79641	0.522	0.9866
Pure error	3	4.5774	1.5258		
Total error	12	11.74512			


**Term**		**Estimate**	**Std error**	***t* Ratio**	**Prob > |t|**

Intercept		98.704355	0.376932	261.86	<0.0001*
Flow rate		0.265	0.312851	0.85	0.4135
Salt concentration at washing step		−4.736	0.312851	−15.14	<0.0001*
Salt concentration at elution step		−0.523	0.312851	−1.67	0.1204
Flow rate*flow rate		−1.457419	0.561898	−2.59	0.0235*
Salt concentration at washing step*salt concentration at washing step		−3.812419	0.561898	−6.87	<0.0001*

*Identified variable with a significant effect on response (*p*-value < 0.05).

%Step yield = 79.6438 – 0.386(flow rate) + 3.541([(NH_4_)_2_SO_4_] at washing step) + 0.53375(flow rate) ([(NH_4_)_2_SO_4_] at washing step) + 3.09125([(NH_4_)_2_SO_4_] at washing step)^2^

%SC content t = 98.70436 + 0.265(flow rate) – 4.736([(NH_4_)_2_SO_4_] at washing step) – 0.523([(NH_4_)_2_SO_4_] at elution step) – 1.457419(flow rate)^2^ – 3.812419([(NH_4_)_2_SO_4_] at washing step)^2^

**FIGURE 3:**
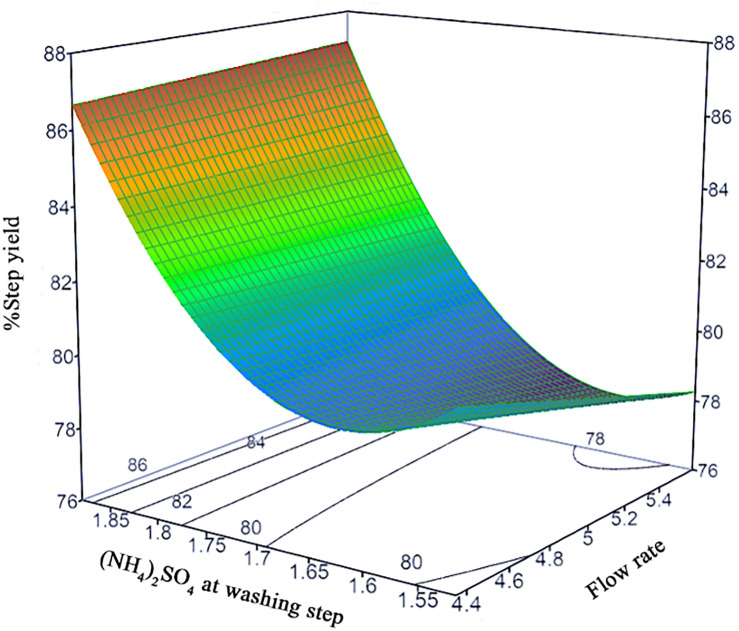
Response surface prediction for %Step yield with (NH_4_)_2_SO_4_ concentration at washing step and flow rate.

**FIGURE 4:**
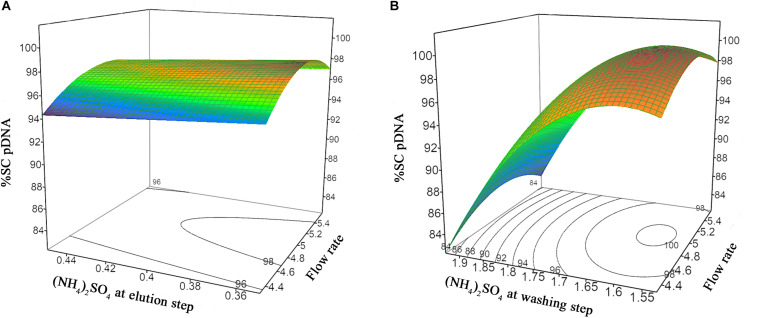
Response surface prediction for %SC pDNA with **(A)** (NH_4_)_2_SO_4_ concentration at washing step and flow rate, **(B)** (NH_4_)_2_SO_4_ concentration at elution step and flow rate.

**FIGURE 5:**
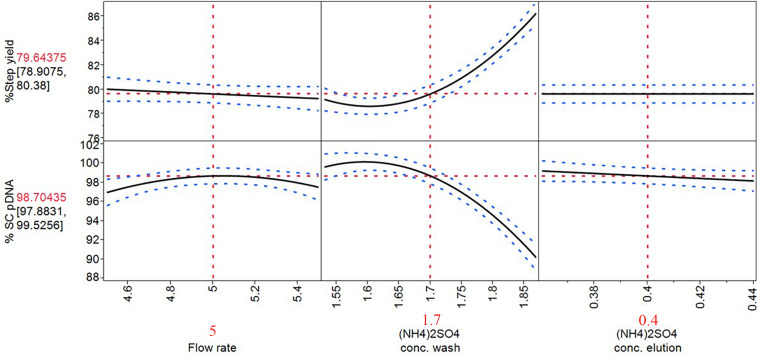
Prediction profiler of optimized process on HIC purification step. The solid lines are the predicted model. The red dashed lines are the interaction plots and the blue dashed lines are the confidence interval.

The prediction models of %Step yield and %SC content were obtained and shown in the quadratic equations. The %Step yield model includes the main effect of flow rate and salt concentration at wash step, the interaction effect of flow rate and salt concentration at washing step, and the quadratic term of salt concentration at washing step. The relationship of the process parameters to the %Step yield response was demonstrated in Figure 3 where different colors, ranging from red, green, to blue, represent different levels of response from high to low. Herein, the negative effect was observed only for the main effect of flow rate though without statistical significance. The positive effects in the main, interaction, and quadratic terms of salt concentration at washing step predominantly and significantly affect the yield. This can be explained by the antichaotropic property of ammonium sulfate. The higher ammonium sulfate concentration at washing step, the more the hydrophobic effects in the solution increase, hence the pDNA remains bound to the column. However, if looking closely at the prediction model of %SC pDNA content, the main and quadratic terms of ammonium sulfate concentration at washing step demonstrated the opposite effect. This %SC prediction model contains the main effect of flow rate, salt concentration at washing step and elution step, and the quadratic term of flow rate and salt concentration at washing step. Figure 4 demonstrates the relationship of process parameters in a surface plot where the redder contour plots reflect a larger response of %SC content. Moreover, the positive effect was observed only for the main effect of flow rate, but this was less pronounced. It was also observed that there were significantly negative influences of salt concentration at washing step in the main and quadratic terms, meaning that a decrease in these terms will allow an increase in the %SC content of eluted products, which is shown in the prediction profiler Figure 5.

With these optimized process parameters on HIC chromatography step as demonstrated in prediction profiler in Figure 5, flow rate at 5 mL/min, [(NH_4_)_2_SO_4_] of 1.7 M at wash step, and 0.4 M at elution step were achieved. The %SC content was improved from the previous step which was around 80% to 98.70% while the step yield was maintained as high as 79.64%.

**FIGURE 6:**
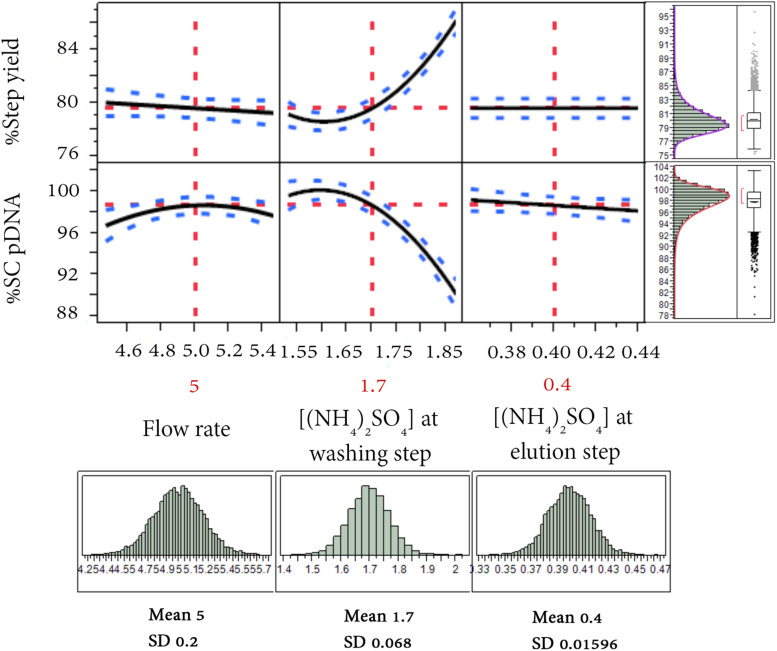
Simulation runs and distribution charts for HIC purification step. The solid lines are the predicted model. The red dashed lines are the interaction plots and the blue dashed lines are the confidence interval.

The tolerance interval analysis is used to investigate the variability of the attributes that potentially represent the probability of being out of specification from batch-to-batch. Therefore, a Monte Carlo simulation approach was employed for the tolerance study. 10,000 runs were simulated with the ranges of operation depicted in Figure 6 for flow rate [(5 ± 0.2) mL/min], ammonium sulfate concentration at washing step [(1.7 ± 0.068) M] and elution step [(0.4 ± 0.0159) M], and the random noise from root mean square error (RMSE) of the obtained predictive models listed in Figure 2 (RMSE = 0.9639 for %Step yield model and = 0.9893 for %SC pDNA model). The result of this simulation was illustrated in a prediction profiler in Figure 6. The distribution plots for all process parameters were normal distribution. The TI study at ∝ = 0.95 was selected. Table 4 summarizes the lower and upper intervals of 77.07–83.22% and 93.99–101.77% for %Step yield and %SC pDNA, respectively. With these intervals, we may be able to set the alert and action limits as well as the DNA vaccine specifications with regards to quality term as %SC content for pVax1/LacZ and its insertion.

**TABLE 4:** Tolerance interval study.

**Tolerance interval**	**Proportion**	**Lower TI**	**Upper TI**	**At alpha**
% Step yield	0.9	77.07	83.22	0.95
% SC pDNA	0.9	93.99	101.77	0.95

The predictive models for HIC purification step of DNA vaccine using pVax1/LacZ as a model has been better understood, however, to carry out the process in order to produce *in vivo* materials, more pDNA characterization, such as endotoxin level, residual host cell DNA, and residual host cell protein, toward the requirements from the regulatory views should be considered. Further experimental runs for a verification of the model would be beneficial and fruitful for future scaling up of DNA production processes. We were able to use the predictive models to explain the effects of process parameters and the optimized ranges of operations in HIC purification step toward the achievement of highest recovery and purity for this pVax1/LacZ plasmid.

Toward QbD process understanding of the purification of DNA vaccines, we have not only conducted an optimization experiment but also explored a systematic approach of using DoE to gain better comprehension on the influence of process parameters to the performance and quality attributes. The relationships of these parameters were acquired and led to the creation of models where the design space of the prediction can be used to ensure that the products are within specifications. This strategy has been of interest in biomanufacturing of pharmaceuticals and vaccines.

## Data Availability Statement

The original contributions presented in the study are included in the article/Supplementary Material, further inquiries can be directed to the corresponding author/s.

## Author Contributions

LH obtained grant support and conducted research and analysis. SN performed the experiments, statistics, and analysis. LH and SN wrote the manuscript. PK revised the manuscript. All authors edited and approved the manuscript.

## Conflict of Interest

The authors declare that the research was conducted in the absence of any commercial or financial relationships that could be construed as a potential conflict of interest.
