# Supplementary material for: Toward QbD Process Understanding on DNA Vaccine Purification Using Design of Experiment
Source: Front Bioeng Biotechnol. 2021 May 12;9:657201. doi: 10.3389/fbioe.2021.657201 (PMC8153680; doi:10.3389/fbioe.2021.657201)
Supplement: Supplementary file 1 [file Data_Sheet_1.PDF]

Supplementary Materials

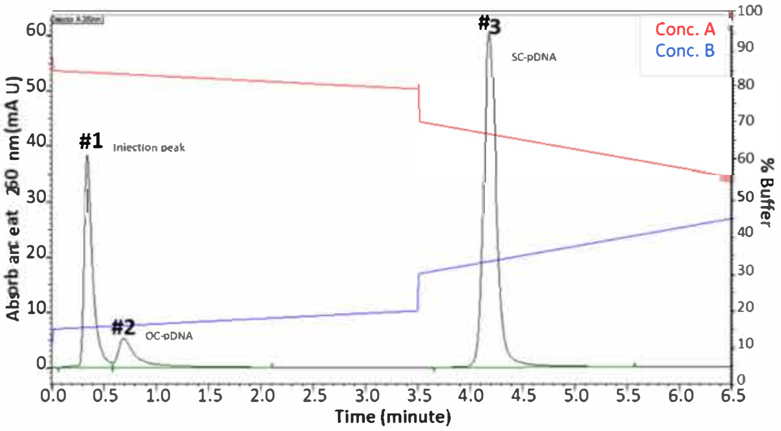

Supplementary Figure 1 HPLC conditions and chormatogram

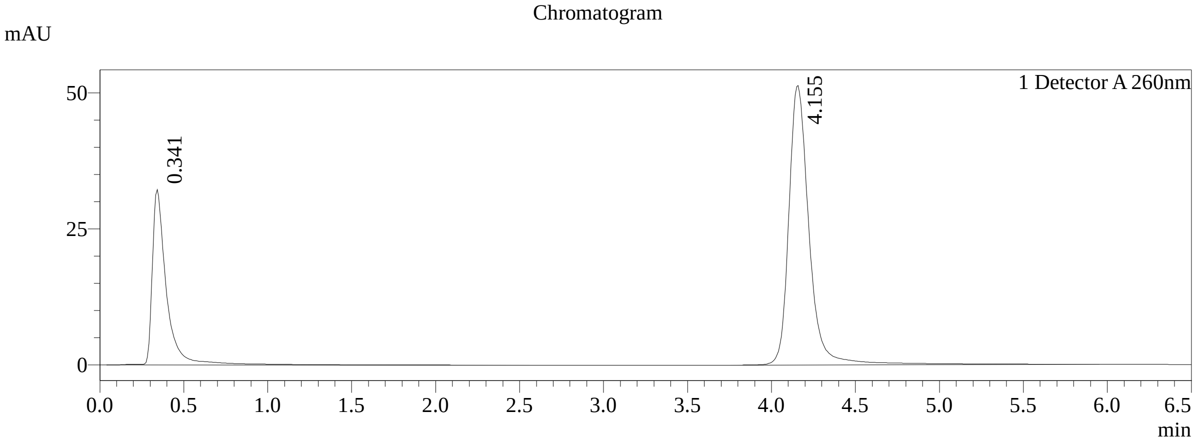

Peak Table

Detector A 260nm

Peak#	Ret. Time	Height	Area	Area%
1	0.341	32262	194411	30.816
2	4.155	51343	436459	69.184
Total		83605	630870	100.000

Supplementary Figure 2 HPLC chromatogram and peak table of HIC elution run number 1

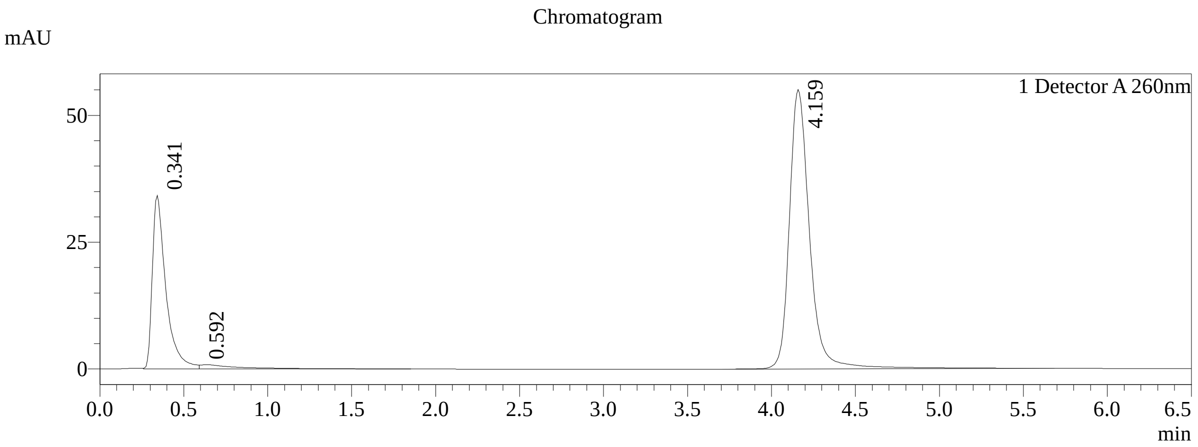

Peak Table

Detector A 260nm

Peak#	Ret. Time	Height	Area	Area%
1	0.341	34223	192942	28.742
2	0.592	785	14115	2.103
3	4.159	55076	464238	69.156
Total		90084	671295	100.000

Supplementary Figure 3 HPLC chromatogram and peak table of HIC elution run number 2

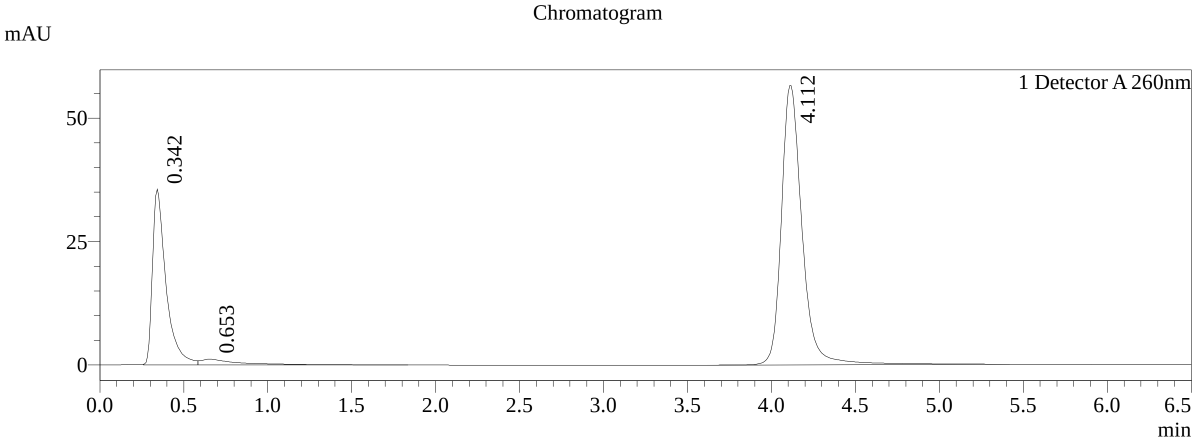

Peak Table

Detector A 260nm

Peak#	Ret. Time	Height	Area	Area%
1	0.342	35620	200792	28.436
2	0.653	1206	20207	2.862
3	4.112	56566	485127	68.703
Total		93392	706127	100.000

Supplementary Figure 4 HPLC chromatogram and peak table of HIC elution run number 3

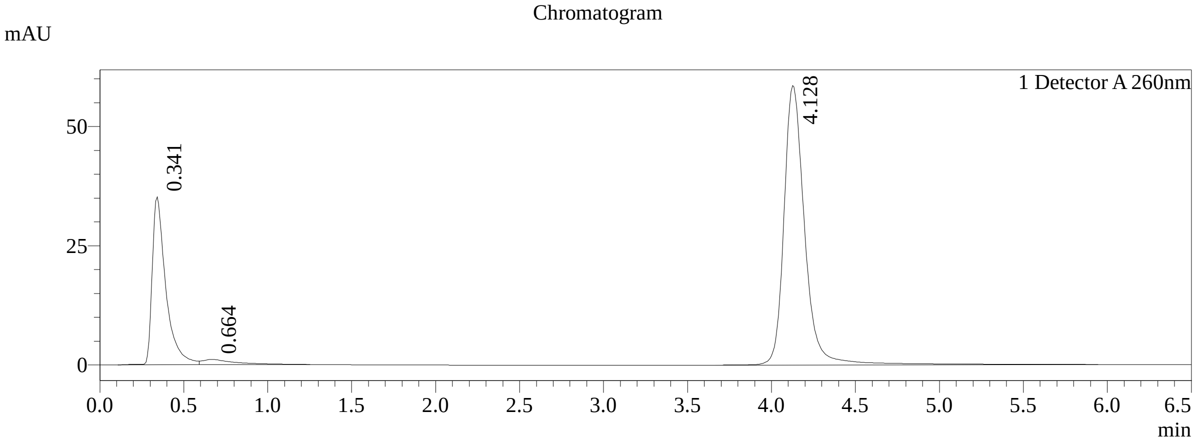

Peak Table

Detector A 260nm

Peak#	Ret. Time	Height	Area	Area%
1	0.341	35250	200531	27.753
2	0.664	1120	15210	2.105
3	4.128	58605	506823	70.142
Total		94975	722564	100.000

Supplementary Figure 5 HPLC chromatogram and peak table of HIC elution run number 4

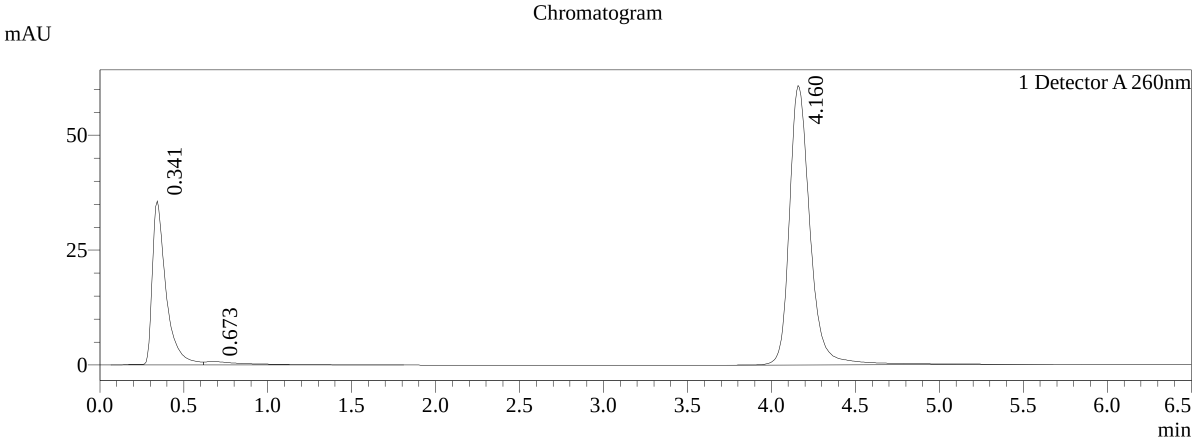

Peak Table

Detector A 260nm

Peak#	Ret. Time	Height	Area	Area%
1	0.341	35660	205033	27.599
2	0.673	753	13808	1.859
3	4.160	60811	524051	70.542
Total		97223	742892	100.000

Supplementary Figure 6 HPLC chromatogram and peak table of HIC elution run number 5

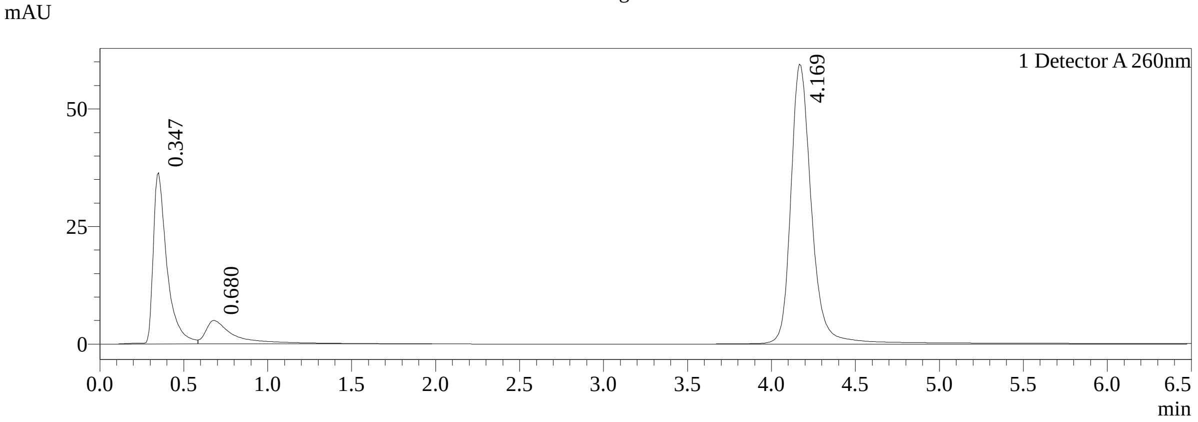

Peak Table

Detector A 260nm

Peak#	Ret. Time	Height	Area	Area%
1	0.347	36431	207741	25.927
2	0.680	4995	61814	7.715
3	4.169	59559	531710	66.359
Total		100985	801266	100.000

Supplementary Figure 7 HPLC chromatogram and peak table of HIC elution run number 6

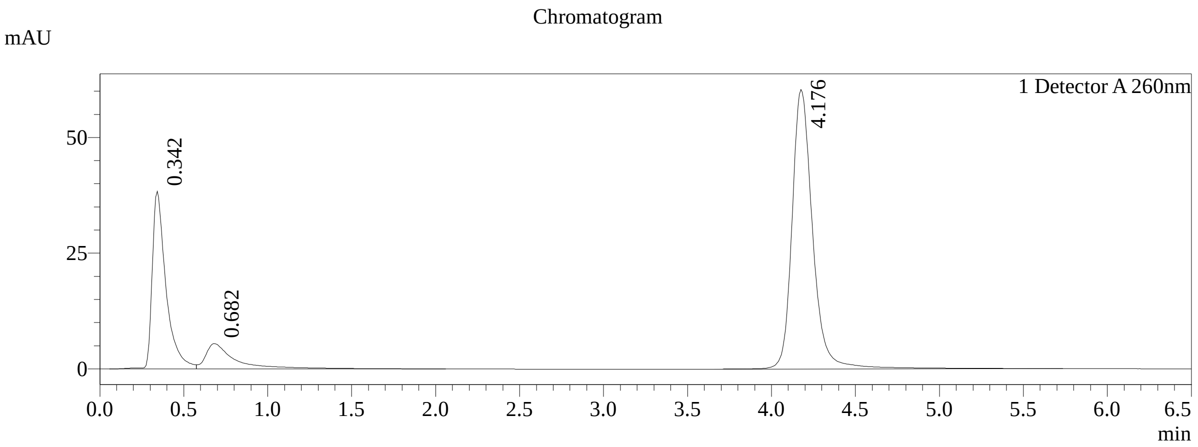

Peak Table

Detector A 260nm

Peak#	Ret. Time	Height	Area	Area%
1	0.342	38413	219922	27.231
2	0.682	5492	68504	8.482
3	4.176	60372	519205	64.287
Total		104277	807631	100.000

Supplementary Figure 8 HPLC chromatogram and peak table of HIC elution run number 7

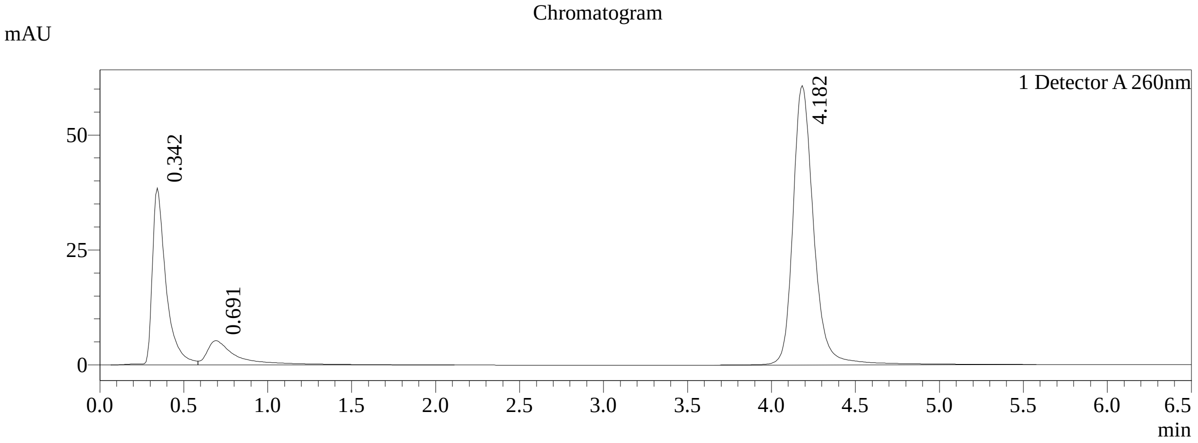

Peak Table

Detector A 260nm

Peak#	Ret. Time	Height	Area	Area%
1	0.342	38487	219223	27.029
2	0.691	5280	67226	8.289
3	4.182	60744	524620	64.682
Total		104510	811070	100.000

Supplementary Figure 9 HPLC chromatogram and peak table of HIC elution run number 8

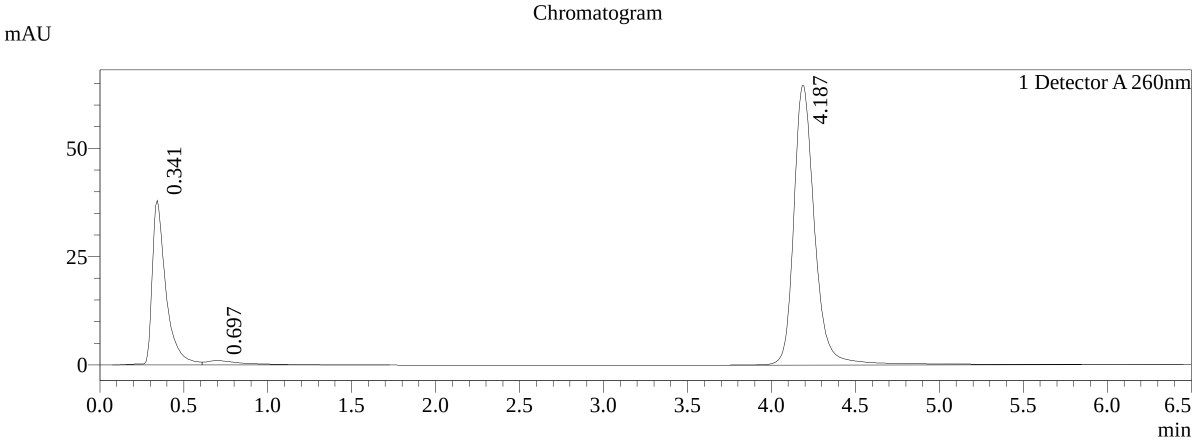

Peak Table

Detector A 260nm

Peak#	Ret. Time	Height	Area	Area%
1	0.341	37993	217694	27.170
2	0.697	1041	17353	2.166
3	4.187	64465	566190	70.665
Total		103498	801237	100.000

Supplementary Figure 10 HPLC chromatogram and peak table of HIC elution run number 9

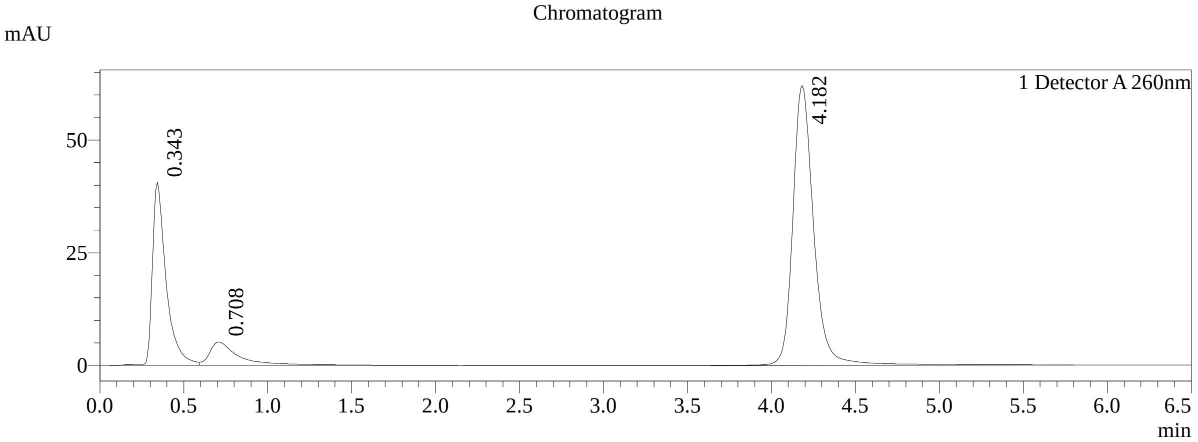

Peak Table

Detector A 260nm

Peak#	Ret. Time	Height	Area	Area%
1	0.343	40607	232440	27.695
2	0.708	5231	66568	7.931
3	4.182	62073	540287	64.374
Total		107911	839295	100.000

Supplementary Figure 11 HPLC chromatogram and peak table of HIC elution run number 10

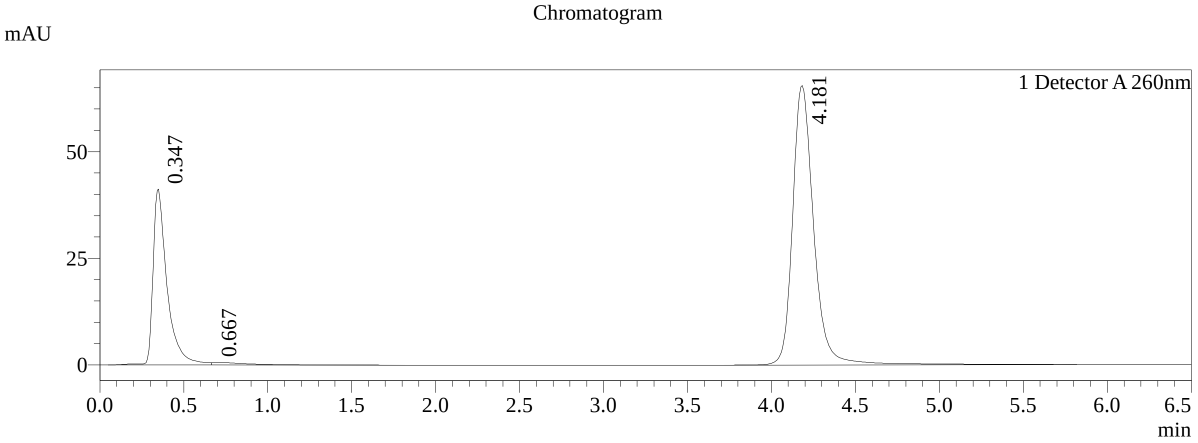

Peak Table

Detector A 260nm

Peak#	Ret. Time	Height	Area	Area%
1	0.347	41224	239839	29.070
2	0.667	522	8568	1.039
3	4.181	65529	576641	69.892
Total		107275	825049	100.000

Supplementary Figure 12 HPLC chromatogram and peak table of HIC elution run number 11

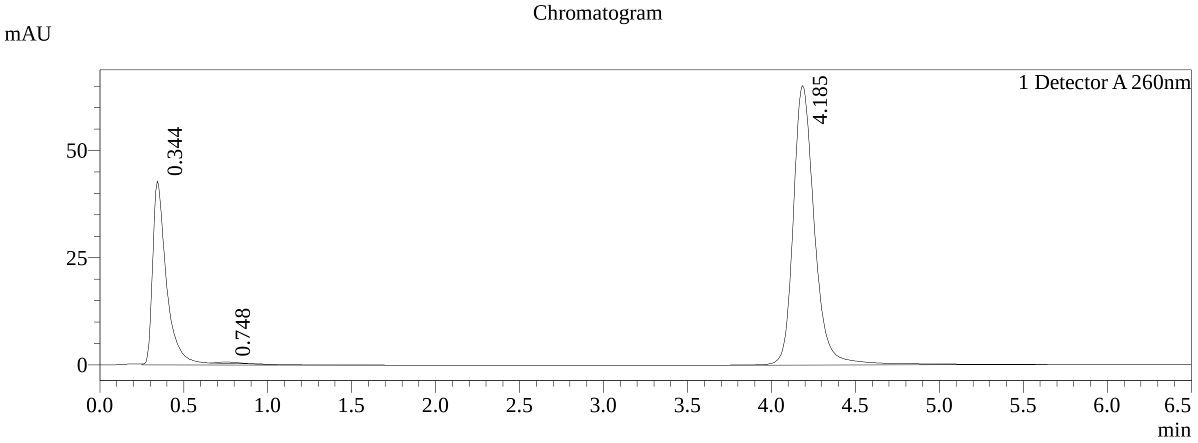

Peak Table

Detector A 260nm

Peak#	Ret. Time	Height	Area	Area%
1	0.344	42862	254803	30.609
2	0.748	295	2552	0.307
3	4.185	65202	575089	69.084
Total		108358	832444	100.000

Supplementary Figure 13 HPLC chromatogram and peak table of HIC elution run number 12

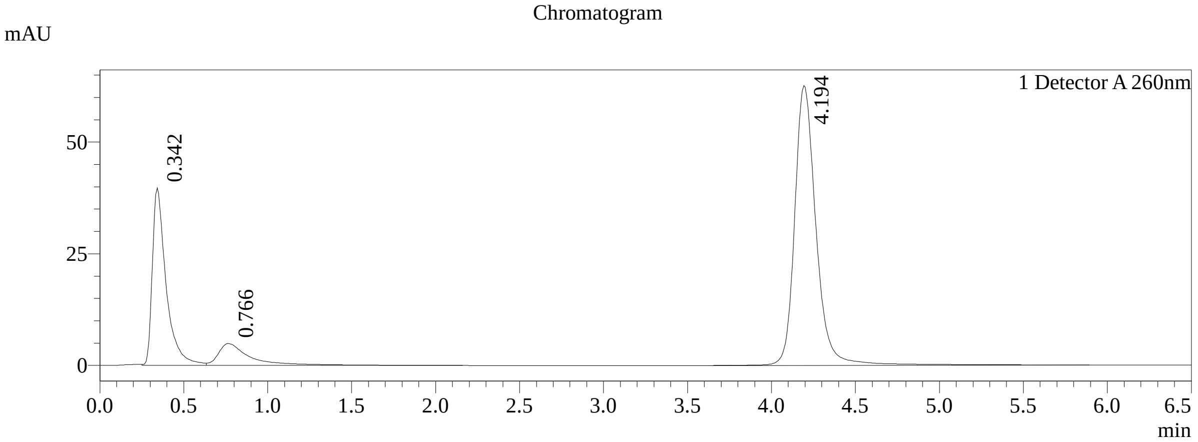

Peak Table

Detector A 260nm

Peak#	Ret. Time	Height	Area	Area%
1	0.342	39776	227649	26.733
2	0.766	4916	66471	7.806
3	4.194	62620	557437	65.461
Total		107312	851558	100.000

Supplementary Figure 14 HPLC chromatogram and peak table of HIC elution run number 13

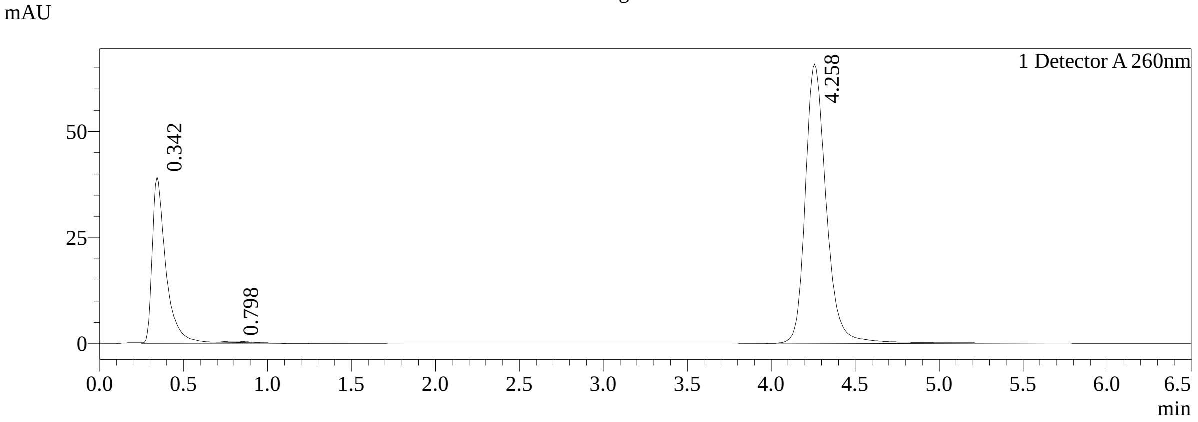

Peak Table

Detector A 260nm

Peak#	Ret. Time	Height	Area	Area%
1	0.342	39291	233836	28.623
2	0.798	333	3250	0.398
3	4.258	65844	579877	70.980
Total		105468	816963	100.000

Supplementary Figure 15 HPLC chromatogram and peak table of HIC elution run number 14

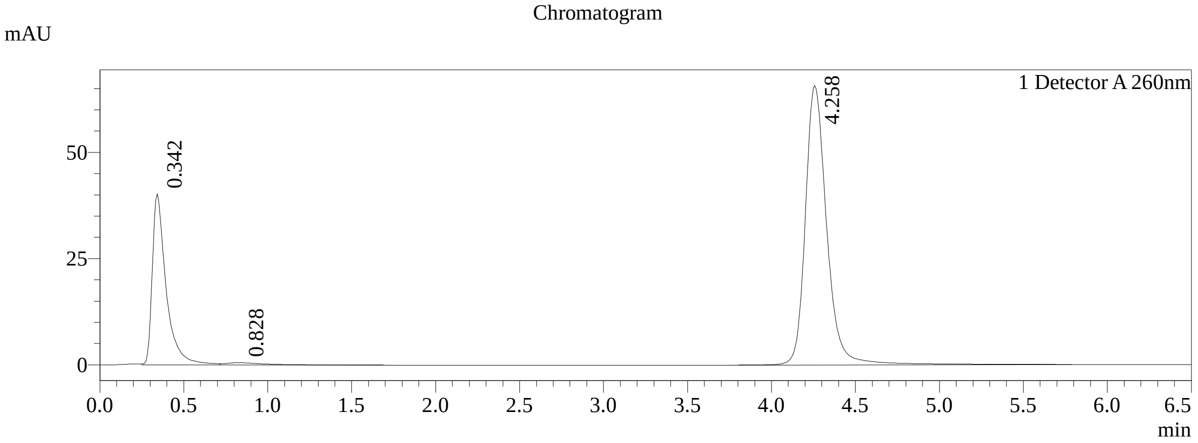

Peak Table

Detector A 260nm

Peak#	Ret. Time	Height	Area	Area%
1	0.342	40174	231214	27.953
2	0.828	540	9266	1.120
3	4.258	65740	586664	70.926
Total		106454	827144	100.000

Supplementary Figure 16 HPLC chromatogram and peak table of HIC elution run number 15

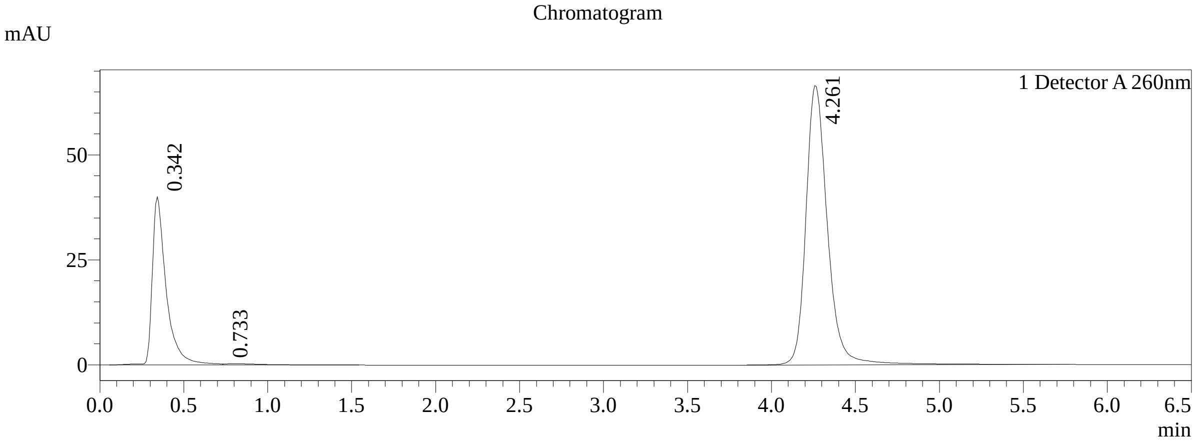

Peak Table

Detector A 260nm

Peak#	Ret. Time	Height	Area	Area%
1	0.342	40006	231314	27.757
2	0.733	262	4268	0.512
3	4.261	66567	597766	71.731
Total		106835	833348	100.000

Supplementary Figure 17 HPLC chromatogram and peak table of HIC elution run number 16

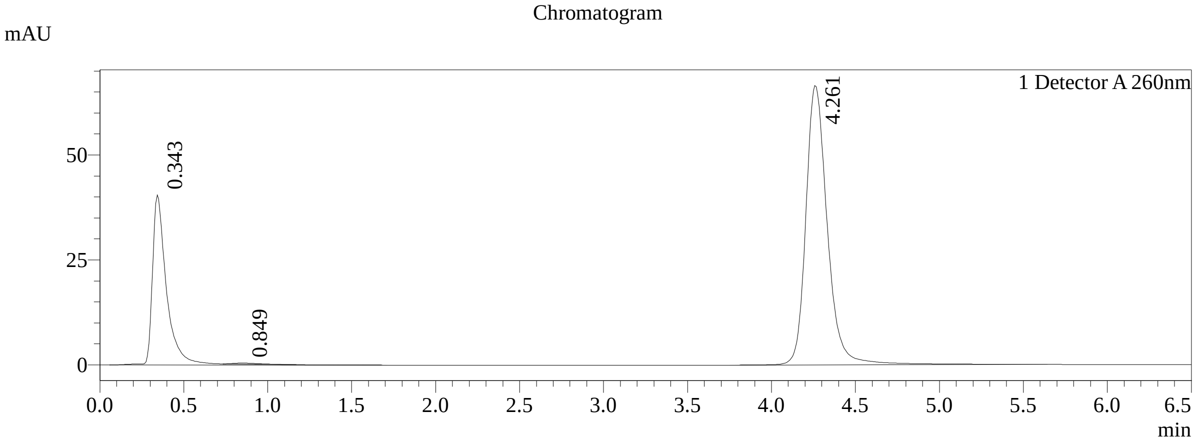

Peak Table

Detector A 260nm

Peak#	Ret. Time	Height	Area	Area%
1	0.343	40472	240046	28.641
2	0.849	237	2533	0.302
3	4.261	66557	595547	71.057
Total		107266	838126	100.000

Supplementary Figure 18 HPLC chromatogram and peak table of HIC elution run number 17

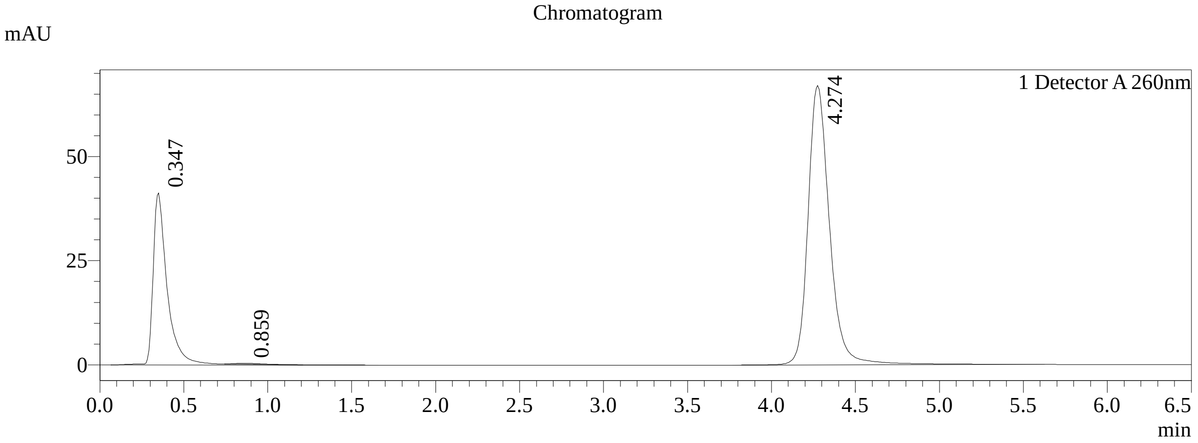

Peak Table

Detector A 260nm

Peak#	Ret. Time	Height	Area	Area%
1	0.347	41261	243924	28.804
2	0.859	230	2449	0.289
3	4.274	67077	600463	70.907
Total		108568	846835	100.000

Supplementary Figure 19 HPLC chromatogram and peak table of HIC elution run number 18
